# Anticoagulant Properties of a Green Algal Rhamnan-type Sulfated Polysaccharide and Its Low-molecular-weight Fragments Prepared by Mild Acid Degradation

**DOI:** 10.3390/md16110445

**Published:** 2018-11-12

**Authors:** Xue Liu, Peng Du, Xiao Liu, Sujian Cao, Ling Qin, Meijia He, Xiaoxi He, Wenjun Mao

**Affiliations:** 1 Key Laboratory of Marine Drugs of Ministry of Education, Shandong Provincial Key Laboratory of Glycoscience and Glycotechnology, School of Medicine and Pharmacy, Ocean University of China, Qingdao 266003, China; wenshan389161833@163.com (X.L.); dp9543@stu.ouc.edu.cn (P.D.); 18435166197@163.com (X.L.); caosujian@stu.ouc.edu.cn (S.C.); ql1599@stu.ouc.edu.cn (L.Q.); hmj@stu.ouc.edu.cn (M.H.); hexiaoxi@ouc.edu.cn (X.H.); 2 Laboratory for Marine Drugs and Bioproducts of Qingdao National Laboratory for Marine Science and Technology, Qingdao 266237, China; 3 Biology Institute, Qilu University of Technology (Shandong Academy of Sciences), Jinan 250103, China

**Keywords:** sulfated polysaccharide, depolymerization, low-molecular-weight fractions, anticoagulant activity, fibrin(ogen)olytic activity, thrombolytic activity

## Abstract

The active sulfated polysaccharide from seaweed possesses important pharmaceutical and biomedical potential. In the study, *Monostroma* sulfated polysaccharide (MSP) was obtained from *Monostroma angicava*, and the low-molecular-weight fragments of MSP (MSP-Fs: MSP-F1–MSP-F6) were prepared by controlled acid degradation. The molecular weights of MSP and MSP-F1–MSP-F6 were 335 kDa, 240 kDa, 90 kDa, 40 kDa, 24 kDa, 12 kDa, and 6.8 kDa, respectively. The polysaccharides were sulfated rhamnans that consisted of →3)-α-l-Rha*p*-(1→ and →2)-α-l-Rha*p*-(1→ units with partial sulfation at C-2 of →3)-α-l-Rha*p*-(1→ and C-3 of →2)-α-l-Rha*p*-(1→. Anticoagulant properties in vitro of MSP and MSP-F1–MSP-F6 were evaluated by studying the activated partial thromboplastin time, thrombin time, and prothrombin time. Anticoagulant activities in vivo of MSP and MSP-F4 were further evaluated; their fibrin(ogen)olytic activities in vivo and thrombolytic properties in vitro were also assessed by D-dimer, fibrin degradation products, plasminogen activator inhibitior-1, and clot lytic rate assays. The results showed that MSP and MSP-F1–MSP-F4 with molecular weights of 24–240 kDa had strong anticoagulant activities. A decrease in the molecular weight of MSP-Fs was accompanied by a decrease in the anticoagulant activity, and higher anticoagulant activity requires a molecular weight of over 12 kDa. MSP and MSP-F4 possessed strong anticoagulant activities in vivo, as well as high fibrin(ogen)olytic and thrombolytic activities. MSP and MSP-F4 have potential as drug or helpful food supplements for human health.

## 1. Introduction

Marine algal polysaccharides are one of the most important active substances that may be a novel source of drugs and helpful food for human health. To date, a lot of sulfated polysaccharides from marine red and brown seaweeds have been studied in terms of their structures and biological activities such as alginate, sulfated galactans, and fucans [1,2]. However, a few limited investigations on sulfated polysaccharides from green seaweeds have been found, especially sulfated rhamnans from the *Monostroma* species. The genus *Monostroma* (Monostromaceae, Chlorophyta) is broadly distributed in coastal waters, and the sulfated rhamnans from the *Monostroma* species have drawn attention [3,4,5].

The sulfated rhamnans from the *Monostroma* species, which are composed of large amounts of α-l-rhamnose, are quite scarce in nature [6,7]. Some investigations have found that the sulfated rhamnans from the *Monostroma* species exhibited anticoagulant, anticancer, immunomodulatory, and antiviral activities [5,8,9,10,11,12]. In particular, the interest in the anticoagulant properties of sulfated rhamnans from the *Monostroma* species is increasing [13,14]. In addition, little information was available on the correlation of molecular weights of the sulfated rhamnans with their anticoagulant activities [15]. The high molecular weight could restrict the pharmaceutical applications of the sulfated polysaccharides, because it is difficult to pass through organizational barriers and then enter the interior of cells or attach to the receptors [16,17,18,19]. It was noted that the molecular weight might have an important effect for the anticoagulant activity of polysaccharides [18,20,21]. A decrease in the molecular weight of (1→3)-β-glucans was accompanied by a decrease in the anticoagulant activity, and the minimal molecular weight had to be over 30 kDa [20]. A similar trend could be also observed on a sulfated fucan from the sea urchin *Lytechinus variegates* [21]. The low molecular weight fractions F-I and F-II from the sulfated galactan of *Botryocladia occidentalis* exhibited high anticoagulant activities, and the total thrombin inhibition mediated by heparin cofactor II was even stronger than the parent polysaccharide [18]. The low-molecular-weight fractions prepared by the partial depolymerization of native polysaccharide represent a source of potential anticoagulants to be explored [22,23,24].

The green seaweed *Monostroma angicava* grows in the upper part of the intertidal zone, and one particularly interesting feature of the seaweed is its richness in polysaccharides. In the present study, *Monostroma* sulfated polysaccharide (MSP) from *M. angicava* was isolated, and its low-molecular-weight fractions MSP-Fs were prepared by the mild acid hydrolysis of MSP. The chemical characteristics and anticoagulant activities in vitro of MSP and MSP-Fs were investigated. Furthermore, the anticoagulant and fibrin(ogen)olytic activities in vivo of MSP and low-molecular-weight fraction MSP-F4 were evaluated, and their thrombolytic properties in vitro were also studied.

## 2. Results and Discussion

### 2.1. Preparation and Chemical Compositions of Monostroma Sulfated Polysaccharide MSP and Its Low-molecular-weight Fractions, MSP-Fs

**Figure 1:**
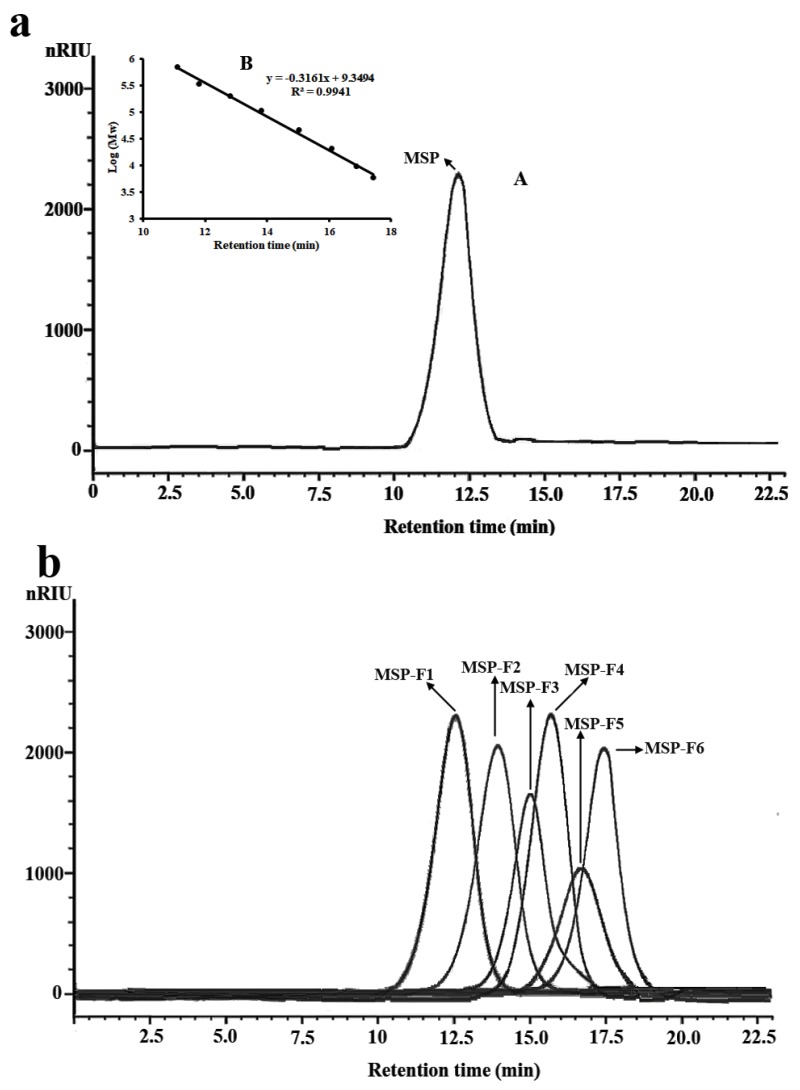
High-performance gel permeation chromatography (HPGPC) chromatograms of *Monostroma* sulfated polysaccharide (MSP) and low-molecular-weight MSP fractions (MSP-Fs). (**a**) HPGPC chromatogram of MSP (A) and the standard curve of molecular weight (B); (**b**) HPGPC chromatograms of MSP-Fs.

The milled alga was extracted twice with ethanol in order to eliminate the phenolic compound or pigment in the dried alga. Then, *Monostroma* sulfated polysaccharide (MSP) was extracted from the alga using distilled water and purified using anion-exchange and gel-permeation chromatography. MSP appeared as a single and symmetrical peak in the high-performance gel permeation chromatography (HPGPC) chromatogram (Figure 1a). The yield of MSP from dry alga (*w*/*w*) was about 6.25%, as shown in Table 1. MSP contained a large amount of rhamnose with a minor amount of xylose. The sulfate content of MSP was high (27.32%), and the molecular weight was 335 kDa. Uronic acid and protein were not found in MSP. The results indicated that MSP had a different chemical component from other sulfated polysaccharides from the *Monostroma* species (3–5,11,13,14,16).

The low-molecular-weight fractions of MSP (named MSP-Fs) were prepared by mild acid hydrolysis. The depolymerization of MSP with trifluoroacetic acid (TFA) was monitored by gel filtration chromatography at different time points. The reaction conditions were optimized to obtain six low-molecular-weight fractions, which were MSP-F1, MSP-F2, MSP-F3, MSP-F4, MSP-F5, and MSP-F6, respectively. The purity of the low-molecular-weight fractions were also detected by HPGPC. As shown in Figure 1b, MSP-F1, MSP-F2, MSP-F3, MSP-F4, MSP-F5, and MSP-F6 gave a single and symmetrical peak in the HPGPC chromatogram, respectively. Therefore, the sulfated polysaccharide fragments could be homogeneous polysaccharides. The molecular weights of MSP-F1, MSP-F2, MSP-F3, MSP-F4, MSP-F5, and MSP-F6 were 240 kDa, 90 kDa, 40 kDa, 24 kDa, 12 kDa, and 6.8 kDa, respectively. As listed in Table 1, the chemical components of MSP-Fs were similar with those of MSP. The results suggested that the method of the acid degradation could successfully break the glycosidic linkages in MSP without destroying the chemical structure of MSP.

### 2.2. Structural Characteristics of MSP

A Fourier-transform infrared (FTIR) spectrum (Figure S1) of MSP showed characteristic bands at 855 cm^−1^, which were attributed to the stretching vibration of C−O−S of sulfate ester, and 1241 cm^−1^ were due to stretching vibration of S=O of the sulfate group [25]. The bands at 3433 cm^−1^ and 2937 cm^−1^ were assigned to the stretching vibration of O−H and the stretch vibration of C−H, respectively. The signal at 1631 cm^−1^ was due to the bending vibration of HOH, while the band at 1051 cm^−1^ was due to the stretching vibration of C−O, and the band at 1450 cm^−1^ was the absorption peak of the variable angle vibration of the C−H bond [13].

Methylation analysis gave important information about the linkage pattern and sulfation position of the sulfated polysaccharide. The results of methylation analysis of MSP and its desulfated product MSP-Ds were listed in Table 2. MSP was primarily composed of (1→3)-linked rhamnose, (1→2)-linked rhamnose, and (1→2,3)-linked rhamnose units. Moreover, →4)-Xyl*p*-(1→ was also detected. Compared with the result of MSP, increased amounts of (1→3)-linked rhamnose and (1→2)-linked rhamnose units were detected, and a decreased quantity of (1→2,3)-linked rhamnose units were observed in MSP-Ds. Thus, the sulfate substitutions were deduced to be at the C-2 of (1→3)-linked rhamnose and C-3 of (1→2)-linked rhamnose units. Moreover, the C-4 sulfation site of Xyl*p*-(1→ could be deduced by the disappearance of →4)-Xyl*p*-(1→ in MSP-Ds. From the data, it was extrapolated that 24.76% of the rhamnose units in MSP was sulfated, specifically, 21.49% of (1→3)-linked rhamnose units and 3.27% of (1→2)-linked rhamnose units.

**Figure 2:**
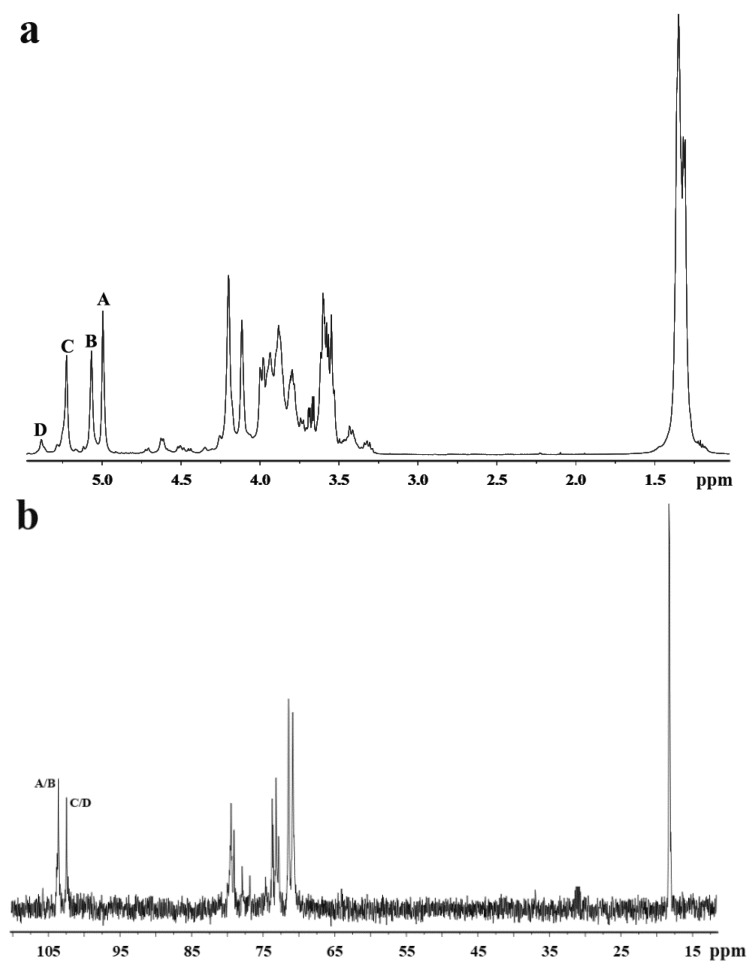

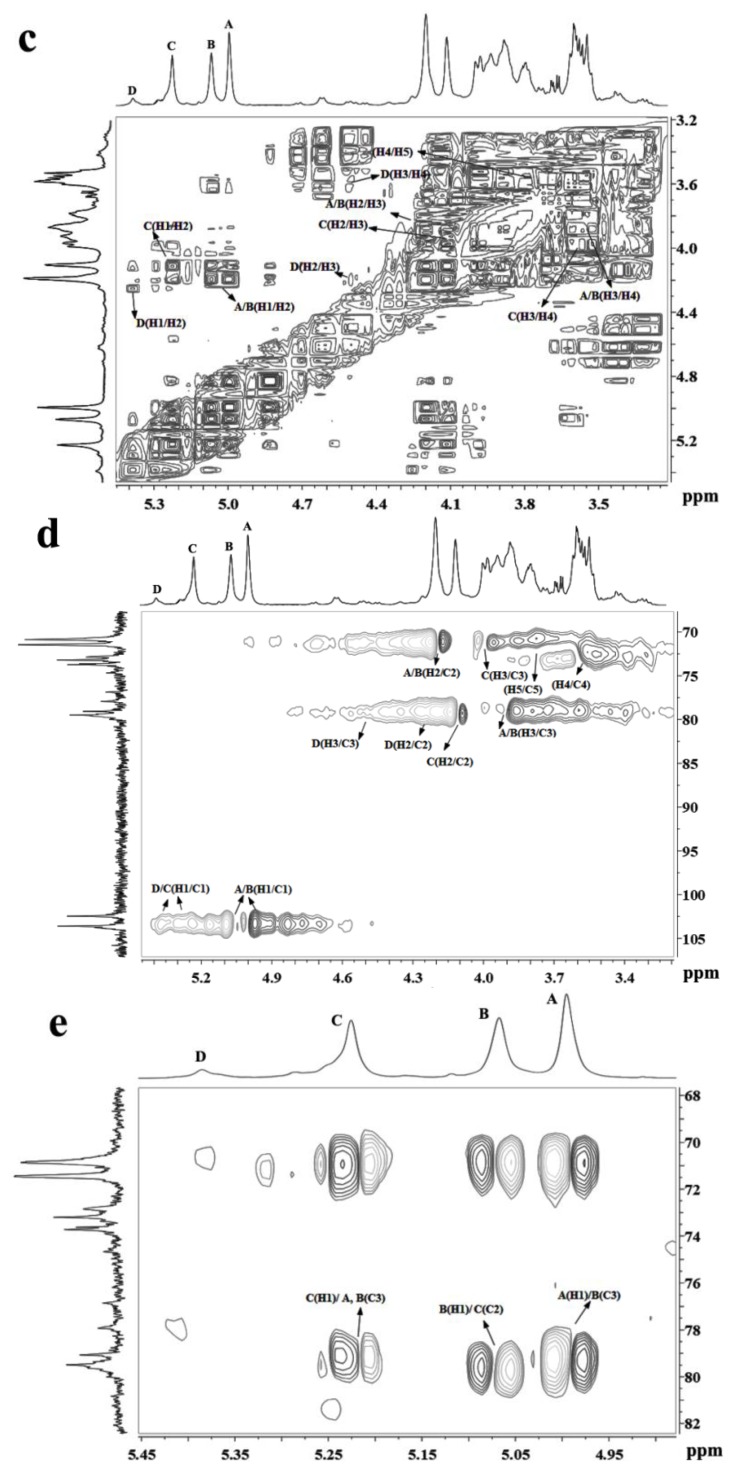
NMR spectra of MSP-Ds. (**a**) ^1^H NMR; (**b**) ^13^C NMR; (**c**) ^1^H–^1^H COSY; (**d**) ^1^H–^13^C HSQC; (**e**) ^1^H–^13^C HMBC. A: →3)-α-l-Rha*p*-(1→; B: →3)-α-l-Rha*p*-(1→; C: →2)-α-l-Rha*p*-(1→; D: →2)-α-l-Rha*p*(3SO_4_)-(1→. Rha*p*: rhamnopyranose.

The ^1^H NMR spectrum of MSP-Ds (Figure 2a) showed four anomeric proton signals at 4.99 ppm, 5.07 ppm, 5.22 ppm, and 5.37 ppm with relative integrals of 1.05:0.97:1.00:0.20, which were attributed to α-l-rhamnopyranose. The signals were labeled A–D from the low to high field. The signals at 4.99 ppm and 5.07 ppm were assigned to the H-1 of 1,3-linked α-l-rhamnopyranose, 5.22 ppm was assigned to the H-1 of 1,2-linked α-l-rhamnopyranose and 5.37 ppm was assigned to the H-1 of 1,2-linked-3-sulfated α-l-rhamnopyranose [13,14]. Besides, the signal at 1.33 ppm was due to the proton of CH_3_ group of the rhamnose unit.

In the anomeric region of the ^13^C NMR spectrum of MSP-Ds (Figure 2b), the main anomeric carbon resonances of α-l-rhamnopyranose occurred at 102–104 ppm. The signals at 70–80 ppm were assigned to C-2–C-5 of the glycosidic rings. The signal at 18.23 ppm was C-6 of the rhamnose unit. The α-anomeric configuration of rhamnopyranose residues was also deduced from H-5 signal at 3.79 ppm and C-5 signal at 70.82 ppm [26]. ^1^H NMR spin systems of MSP-Ds were assigned by the ^1^H−^1^H correlated spectroscopy (COSY) spectrum (Figure 2c). Combined with the analysis of the ^1^H−^13^C heteronuclear single quantum coherence spectroscopy (HSQC) spectrum (Figure 2d), the assignment of all of the signals of the major units could be completed. ^1^H and ^13^C NMR shifts of MSP-Ds are listed at Table 3. The ^1^H−^13^C heteronuclear multiple-bond correlation (HMBC) spectrum (Figure 2e) was used to further assign the chemical shifts of the main spin system. The anomeric proton signal of A was related to the C-3 of B, while the H-1 of B was correlated with the C-2 of C. Sugar sequences →3)-α-l-Rha*p*-(1→3)-α-l-Rha*p*-(1→, →3)-α-l-Rha*p*-(1→2)-α-l-Rha*p*-(1→ were deduced. The sequence →2)-α-l-Rha*p*-(1→3)-α-l-Rha*p*-(1→ was also deduced by the crossing signals C(H-1)/A, B(C-3).

The ^1^H NMR spectrum of MSP (Figure S2a) showed four anomeric proton signals at 5.07 ppm, 5.25 ppm, 5.35 ppm, and 5.50 ppm with relative integrals of 1.44:1.00:0.38:0.83. The signals were labeled A–D from low to high field. The chemical shifts from 5.50 ppm to 5.07 ppm were attributed to α-l-rhamnopyranose units. Compared with the ^1^H NMR spectrum of MSP-Ds, a new signal at 5.50 ppm was observed in the ^1^H NMR spectrum of MSP. In view of all of the data from the ^1^H NMR, ^13^C NMR (Figure S2b), ^1^H–^1^H COSY (Figure S2c), ^1^H–^13^C HSQC (Figure S2d), and the ^1^H–^1^H nuclear Overhauser enhancement spectroscopy (NOESY) (Figure S2e) spectra, the signal of D at 5.50 ppm was assigned to the (1→3)-linked-2-sulfated-α-l-rhamnopyranose. Also, 5.07 ppm was assigned to the H-1 of 1,3-linked α-l-rhamnopyranose, 5.25 ppm was ascribed to the H-1 of 1,2-linked α-l-rhamnopyranose, and 5.35 ppm was attributed to the H-1 of 1,2-linked-3-sulfated α-l-rhamnopyranose. In the ^13^C NMR spectrum of MSP, the main anomeric carbon resonances of α-l-rhamnopyranose at 100–104 ppm and C-2–C-5 signals at 70–80 ppm were found. Besides, the signal at 105.86 ppm was due to the non-reducing terminal β-d-xylosyl units [14]. ^1^H and ^13^C NMR shifts (Table 4) of MSP were assigned by the ^1^H–^1^H COSY and ^1^H–^13^C HSQC. From the ^1^H–^1^H NOESY spectrum, the strong crossing signals A(H1)/D(H3) and D(H1)/A(H3) indicated the repeat unit →3)-α-l-Rha*p*-(1→3)-α-l-Rha*p*(2SO_4_)-(1→. Moreover, the H-1 of units B and C were associated with the H-3 of A, the linkages →2)-α-l-Rha*p*-(1→3)-α-l-Rha*p*-(1→ and →2)-α-l-Rha*p*(3SO_4_)-(1→3)-α-l-Rha*p*-(1→ were suggested. The presence of cross signal B(H1)/D(H3) illustrated the sequence →2)-α-l-Rha*p*-(1→3)-α-l-Rha*p*(2SO_4_)-(1→. Besides, the presence of a weak signal of A(H1)/B(H2) indicated that a small amount of sequence →3)-α-l-Rha*p*-(1→2)-α-l-Rha*p*-(1→ might exist. The main disaccharide units could be suggested (Figure S3).

### 2.3. Structural Characteristics of MSP-Fs

As listed in Figure S1, the band at 855 cm^−1^ was assigned to stretching vibration of the C−O−S of sulfate in the axial position, and 1241 cm^−1^ was attributed to the stretching vibration of the S−O of sulfate. The band at 3433 cm^−1^ was the stretching vibration of the OH group, and the signal at 2937 cm^−1^ was assigned to the stretch vibration of the C−H bond. The FT-IR spectra of MSP-Fs were in agreement with that of the MSP, suggesting no main changes of functional groups during acid degradation. The chemical structures of MSP-Fs were similar to those of MSP.

**Figure 3:**
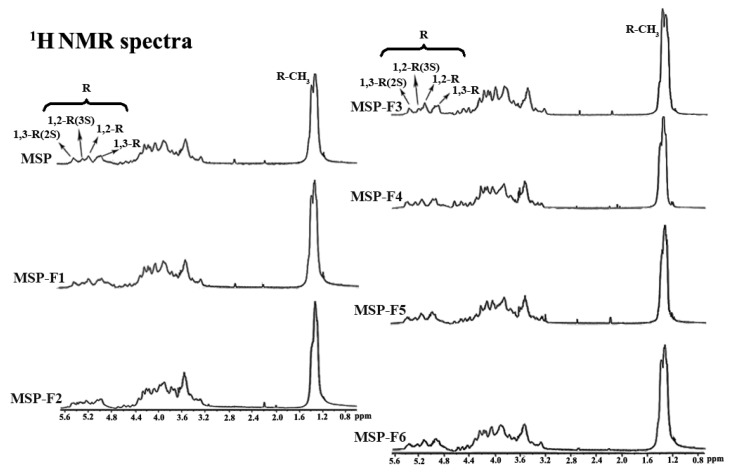
^1^H NMR spectra of MSP and MSP-Fs.

The structural uniformity of MSP and MSP-Fs were further detected by ^1^H NMR spectra (Figure 3). A comparison analysis of ^1^H NMR spectra for MSP and MSP-Fs showed consistency. Four lower-field signals at 5.07 ppm, 5.25 ppm, 5.35 ppm, and 5.50 ppm represented anomeric proton signals of α-l-rhamnopyranose. In detail, 5.07 ppm was assigned to the H-1 of 1,3-linked α-l-rhamnopyranose, 5.25 ppm was assigned to the H-1 of 1,2-linked α-l-rhamnopyranose, 5.35 ppm was assigned to the H-1 of 1,2-linked-3-sulfated α-l-rhamnopyranose and 5.50 ppm was assigned to H-1 of 1,3-linked-2-sulfated α-l-rhamnopyranose [13,14]. Besides, 1.35 ppm was assigned to be the proton of the CH_3_ group of the rhamnose unit. Compared with the results for MSP, the peaks of protons and their relative integrals had no changes. The results demonstrated that the chemical structures of MSP-Fs were similar to that of MSP, suggesting that the acid degradation could cleave the glycoside bond in MSP, but the backbone of the polysaccharide wasn’t destroyed.

### 2.4. Anticoagulant Activities In Vitro of MSP and MSP-Fs

**Figure 4:**
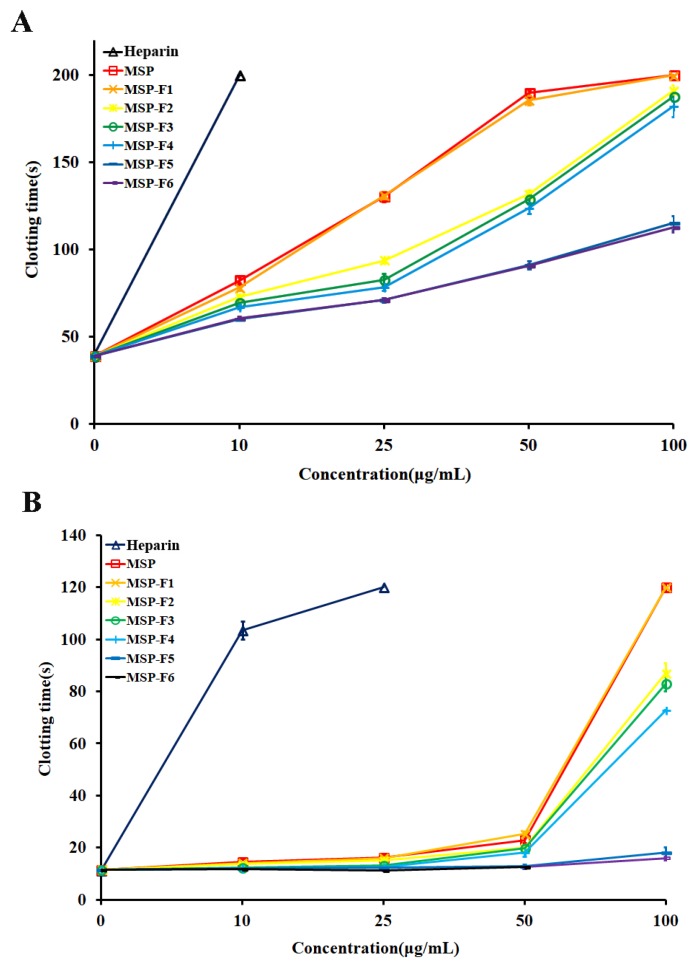

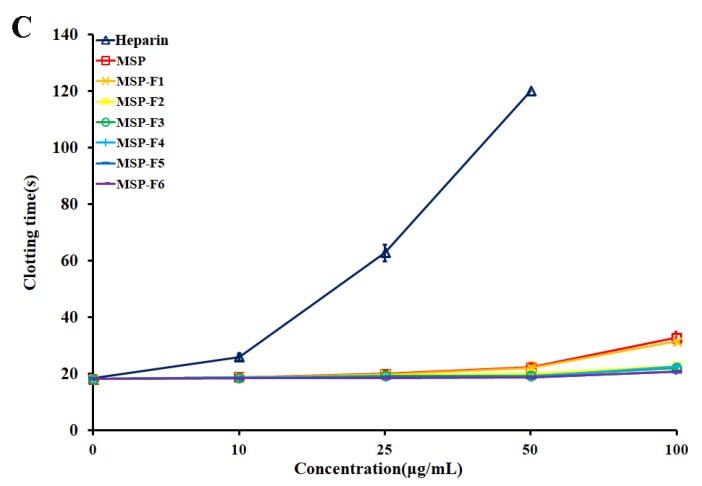
Anticoagulant activities in vitro by APTT, TT, PT assays on MSP and MSP-Fs. (**A**) APTT; (**B**) TT; (**C**) PT.

From Figure 4A, the activated partial thromboplastin time (APTT) activities of MSP and MSP-Fs were concentration-dependent. A significant increase in clotting time was observed from 10 μg/mL to 100 μg/mL of the polysaccharide. The polysaccharides with higher molecular weight (MSP, MSP-F1–MSP-F4) possessed strong APTT activity. The APTT activity of MSP-F1 was similar to that of MSP, and the clotting time was more than 200 s at 100 µg/mL. The APTT activity reduced with the decreasing of molecular weight of the polysaccharides (MSP-F2–MSP-F4). The polysaccharide fractions with lower molecular weight (MSP-F5 and MSP-F6) had markedly lower anticoagulant activities than MSP, especially MSP-F6. The prolongation of APTT indicates an inhibition of the intrinsic and/or common pathways of the coagulation cascade.

From Figure 4B, it was observed that the thrombin time (TT) activities of MSP and MSP-Fs were also concentration-dependent. The polysaccharides with higher molecular weight (MSP, MSP-F1–MSP-F4) exhibited strong TT activities, and the results were similar to those of the APTT activities. MSP-F1 effectively prolonged TT and was similar to MSP. The clotting time of MSP-F1 was more than 120 s at 100 µg/mL. The TT activity reduced with the decreasing of molecular weight of the polysaccharides (MSP-F2–MSP-F4). MSP-F5 and MSP-F6 exhibited markedly lower TT activities than MSP and MSP-F2–MSP-F4. The prolongation of TT suggests an inhibition of thrombin activity or fibrin polymerization.

MSP and MSP-Fs failed to prolong prothrombin time (PT) as the extrinsic pathway of coagulation at the concentrations used in the experiment (Figure 4C). No prolongation of PT demonstrates no inhibition of the extrinsic pathway of coagulation.

In addition, it was observed that the anticoagulant activities of MSP and MSP-Fs were noticeably distinguished from that of heparin. The APTT activities of MSP-F1–MSP-F4 slowly increased, while the APTT activity of heparin rapidly raised, and the clotting time was over 200 s at 10 µg/mL. A similar trend could be also observed on the TT activity. The results suggested that MSP-F1–MSP-F4 may have a minor bleeding hazard than heparin at the same doses, and could be a hopeful antithrombotic agent [27].

It was noted that the polysaccharides with different molecular weights showed different anticoagulant activity. MSP-F1–MSP-F4 with molecular weights of 24–240 kDa had high anticoagulant activities. However, a decrease in the molecular weight of MSP-Fs was accompanied by a decrease in the anticoagulant activity, and a higher anticoagulant activity requires a molecular weight of over 12 kDa. The results suggested that the interaction of sulfated rhamnan with coagulation inhibitors and their target proteases was significantly influenced by the molecular weight. For the sulfated rhamnan, longer chains were required to complete thrombin inhibition. The structure–anticoagulant relationship of the sulfated rhamnan is complicated, and requires in-depth investigation. The correlation of anticoagulant activities of galactans and fucans with their chemical structures has been widely reported [8,28,29,30]. Each type of polysaccharide could form a unique complex with the plasma inhibitor and the target protease. The rhamnan-type sulfated polysaccharides express anticoagulant activity not merely as a function of charge density [31]. Further knowledge on the chemical structures of the sulfated polysaccharides is important for understanding their biological activities.

Based on the results of the in vitro anticoagulant activities of the sulfated polysaccharides, MSP and MSP-F4 were chosen for further in vivo anticoagulant and fibrin(ogen)olytic activities, and in vitro thrombolytic activities assays. Here, MSP-F4 is a representative of low-molecular-weight fractions with high anticoagulant activity in vitro.

### 2.5. Anticoagulant and Fibrin(ogen)olytic Activities In Vivo of MSP and MSP-F4

**Figure 5:**
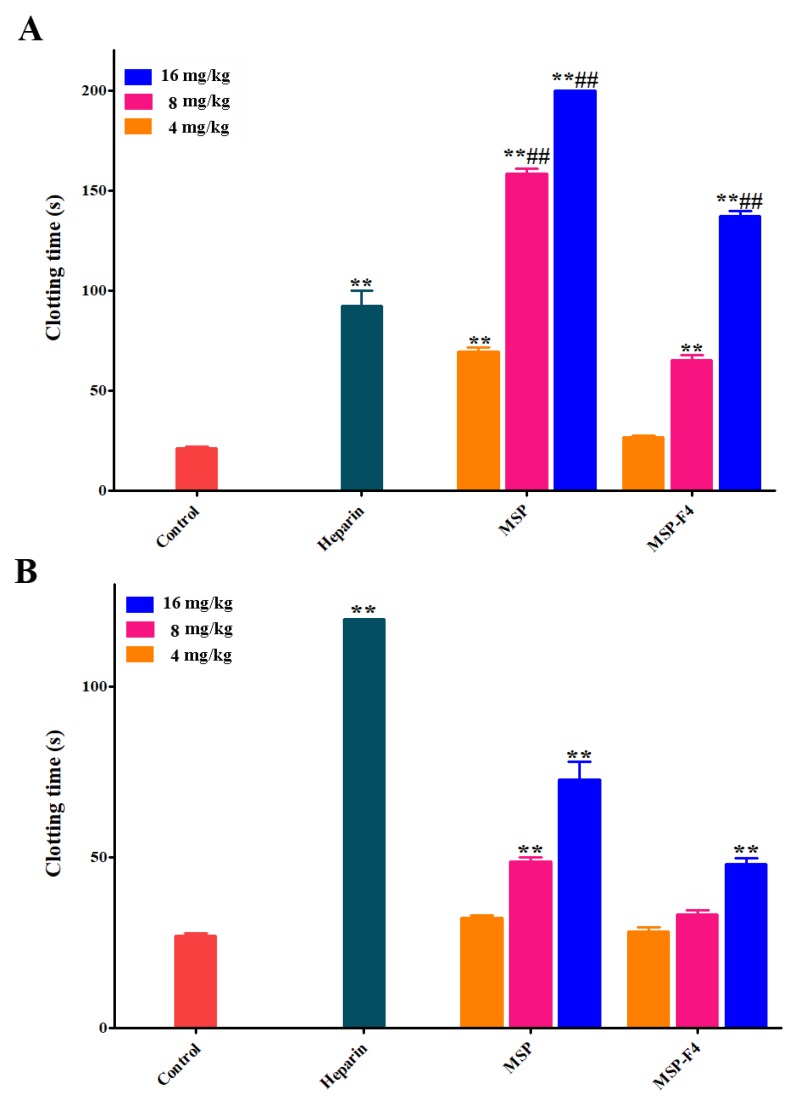

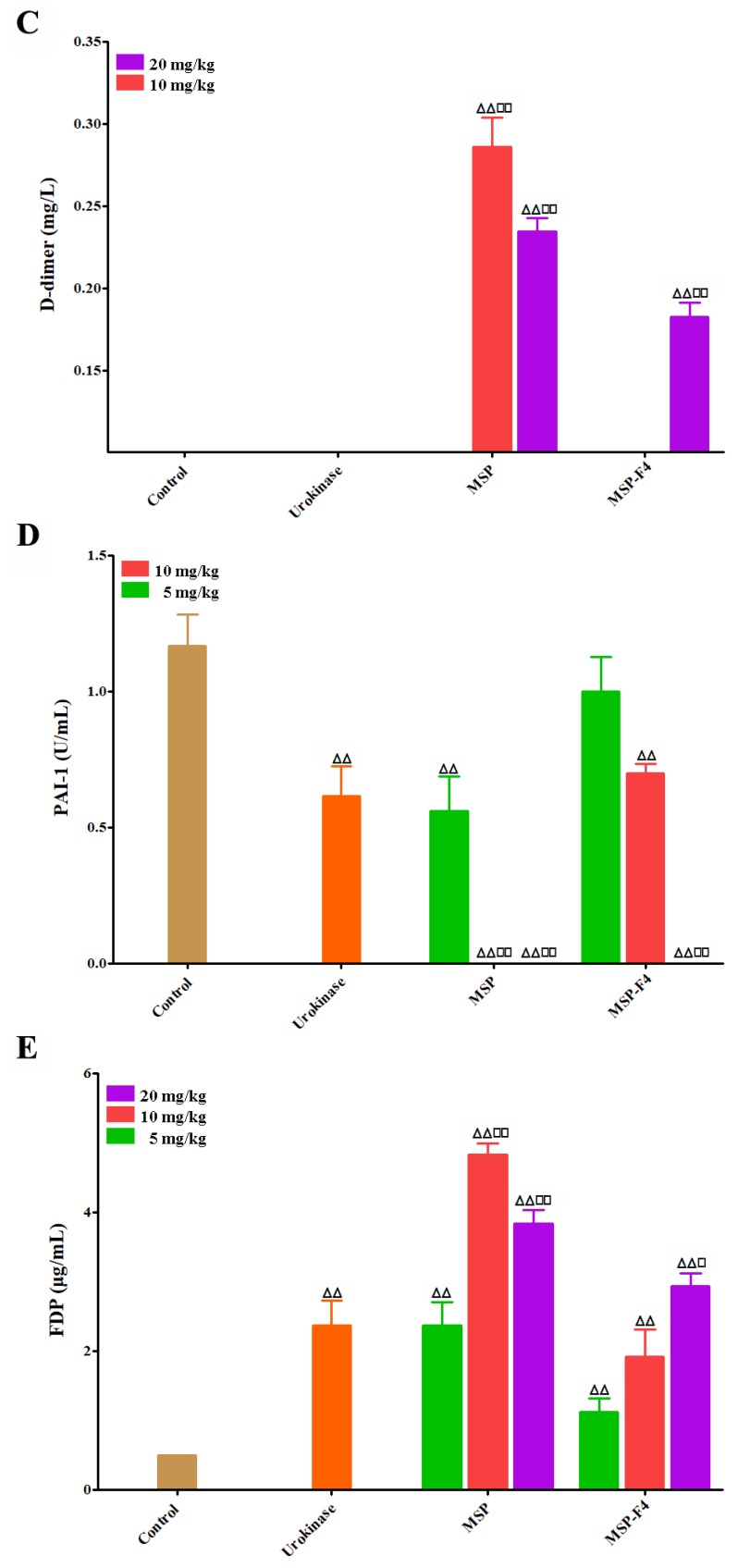
Anticoagulant and fibrin(ogen)olytic activities in vivo of MSP and MSP-F4. (**A**) APTT; (**B**) TT; (**C**) D-dimer, the D-dimer levels of control, urokinase, MSP at 5 mg/kg and MSP-F4 at 5 mg/kg as well as 10 mg/kg groups were below detection limit in this assay; (**D**) Plasminogen activator inhibitior-1 (PAI-1), the PAI-1 value of MSP at 10/20 mg/kg and MSP-F4 at 20 mg/kg was up to 0; (**E**) Fibrin(ogen) degradation products (FDP). Statistical significance: for the anticoagulant activity assay, where ** and ^##^ represented *p* < 0.01; for the fibrin(ogen)olytic and thrombolytic activity assays, where **^□^** represented *p* < 0.05, **^△△^** and **^□□^** represented *p* < 0.01.

The results of anticoagulant activities in vivo of MSP and MSP-F4 are listed in Figure 5A,B. The anticoagulant activities of MSP and MSP-F4 were also dose–dependent. The APTT was strongly prolonged by MSP, and the clotting time was more than 200 s at 16 mg/kg (Figure 5A). MSP-F4 could also significantly prolong the APTT, although the prolongation effect was weaker than that of MSP. A similar trend could be also observed on the TT activity (Figure 5B). In addition, the lack of prolongation effects of MSP and MSP-F4 on the PT was observed (data not shown). It was found that the APTT activities by MSP at 8 mg/kg and 16 mg/kg and MSP-F4 at 16 mg/kg were considerably stronger than that of heparin. However, the TT activities by MSP and MSP-F4 were lower than that of heparin.

Fibrin(ogen)olytic activities in vivo of MSP and MSP-F4 were also assayed by D-dimer, plasminogen activator inhibitior-1 (PAI-1), and fibrin(ogen) degradation products (FDP) [32,33]. Urokinase was used as a positive control. Fibrinogen is the precursor of fibrin. The term ‘fibrin(ogen)olytic’ is more accurate than fibrinolytic or fibrinogenolytic. Thus, the term ‘fibrin(ogen)olytic’ was used in this paper. D-dimer is produced from crosslinked fibrin by the action of plasmin, and contains two crosslinked D fragments of the fibrin protein. Compared with the control group, the levels of D-dimer were noticeably enhanced by MSP and MSP-F4 (Figure 5C). The increasing effects of MSP at 10 mg/kg and 20 mg/kg, and MSP-F4 at 20 mg/kg were markedly higher than that of urokinase, suggesting that MSP and MSP-F4 had strong fibrinolytic and thrombolytic activities. PAI-1 could inhibit the tissue type plasminogen activator and work as a primary regulator of the fibrinolytic system [33]. Compared with the control group, the level of PAI-1 was effectively reduced by MSP and MSP-F4 (Figure 5D). At the dose of 10 mg/kg and 20 mg/kg of MSP and 20 mg/kg of MSP-F4, the PAI-1 level dropped to 0 U/mL. FDP is a small protein fragment that is present in the blood after a blood clot is degraded by fibrinolysis. Compared with the control group, a significant increase in the level of FDP was found with the treatment of MSP and MSP-F4 (Figure 5E). The enhancing effect of MSP at 10 mg/kg reached the maximum. MSP-F4 showed a different trend, and the FDP level enhanced as the dose increased. The increasing effects of MSP at 10 mg/kg and 20 mg/kg, as well as MSP-F4 at 20 mg/kg on FDP, were obviously higher than those of urokinase.

The above results demonstrated that MSP and MSP-F4 had strong fibrin(ogen)olytic properties. It was noted that the fibrin(ogen)olytic activity of MSP-F4 was lower than that of the native polysaccharide MSP. The result suggested that molecular weight had a significant role for the fibrin(ogen)olytic activity of sulfated rhamnan. However, the correlation of the fibrin(ogen)olytic activities of sulfated rhamnans and their molecular weights requires further study.

It was found that MSP had a higher diminishing effect on PAI-1 than PF2, and was similar with Ls2-2 and SPm. Moreover, MSP exhibited stronger rising effects on D-dimer and FDP, and the increase in the effects of MSP was similar with those of PF2 and SPm [13,14,31]. MSP had distinguished structural characteristics from PF2, Ls2-2, and SPm although the polysaccharides were all sulfated rhamnans. The structure-fibrin(ogen)olytic activity relationships of sulfated rhamnans need to be further investigated.

### 2.6. Thrombolytic Activities In Vitro of MSP and MSP-F4

The assay of clot lytic rate was used to evaluate the thrombolytic activities in vitro of MSP and MSP-F4. Urokinase was used as the reference. As listed in Table 5, the clot lytic rate increased as the concentration increasing. Furthermore the dose of MSP-F4 at 5 mg/mL, the clot lytic rate of MSP and MSP-F4 at other concentrations obviously increased compared with the control group. The clot lytic rate of MSP at 20 mg/mL was markedly stronger than that of urokinase. The results demonstrated that MSP and MSP-F4 had high thrombolytic properties in vitro. However, MSP-F4 showed a lower clot lytic rate than MSP, suggesting that the molecular weight might have an important effect on the clot lytic rate. In addition, it was noted that MSP had a higher clot lytic rate than PF2, and it was similar with Ls2-2, but slightly lower than SPm [13,14,31]. The results suggested that the thrombolytic activity of the sulfated rhamnan was closely related to its structure. Further study in thrombolytic activity of the sulfated rhamnan is required.

So far, the investigation on anticoagulant sulfated rhamnans is still low compared with those of anticoagulant sulfated galactans and fucoidans. If more efforts are conducted, more anticoagulant properties on the sulfated rhamnans should be obtained. Recently, algal anticoagulant polysaccharides have provoked more attention, due to heparin being associated with various side effects and the incidence of prion-related diseases. The homogenous polysaccharide, sulfated rhamnan, could be a potential alterative source of heparin. The present results showed that MSP and MSP-F4 exhibited strong anticoagulant activities in vitro and in vivo, as well as high fibrin(ogen)olytic and thrombolytic activities. MSP and MSP-F4 have prospective as drug or food supplements for human health. Continuous efforts in this field will lead to developments of algal-derived agents for clinical uses.

## 3. Materials and Methods

### 3.1. Materials

*M. angicava* was collected from the coastal area of the Yellow Sea in Qingdao, China on June 2013, which is in the growth mature period of the seaweed. The sample is green and 4–9 cm in size. After washing thoroughly in sea water, the sample was dried. Pullulan standards (*Mw*: 5.9 kDa, 9.6 kDa, 21.1 kDa, 47.1 kDa, 107 kDa, 200 kDa, 344 kDa, and 708 kDa) were purchased from Showa Denko K.K. (Tokyo, Japan). Sephacryl S-400/HR and Sephacryl S-300/HR were purchased from GE Health care Life Sciences (Piscataway, NJ, USA). Dialysis membrane (molecular weight cut-off 1000, 3500) was purchased from Lvniao (Yantai, China). l-rhamnose, l-arabinose, d-xylose, d-mannose, d-galactose, d-glucose, d-glucuronic acid, and heparin were purchased from Sigma-Aldrich Chemical Co. (St. Louis, MO, USA). APTT, TT and PT assay reagents were from MD Pacific (Tianjin, China). An FDP kit was purchased from BIOLINKS CO., LTD. (Tokyo, Japan). PAI-1 and D-dimer kits were purchased from Simens Healthcare Diagnostics Products (Marburg, Germany).

### 3.2. Animals

Male Sprague-Dawley (SD) rats with 220–250 g body weight were housed in standard housing conditions with a 12-h light/dark cycle. All of the rats had free access to water, except during test sessions. The male SD rats were the subjects of anticoagulant fibrin(ogen)olytic properties in vivo of MSP and MSP-F4. Animal experiments were approved by the institutional animal care and use committee of the Ocean University of China (OUC-YY-201706001).

### 3.3. Extraction and Purification of MSP

The milled alga (100 g) was extracted twice using ethanol (20 g /L) at 20 °C for 3 h. The residue from alcohol extraction was dispersed in 30 volumes of distilled water and kept at 20 °C for 1 h. The pellet was homogenized (1:30 alga: water) and refluxed at 100 °C for 2 h. The supernatant was collected by centrifugation, dialyzed in a cellulose membrane (molecular weight cut-off 3500), and a crude polysaccharide (19.08 g) in the non-dialyzable fraction was obtained. The material was fractionated by a Q Sepharose Fast Flow column (30 cm × 3.5 cm, GE Healthcare Life Sciences, Piscataway, NJ, USA). The polysaccharide was eluted with 0 mol/L NaCl, 0.75 mol/L NaCl, 1.5 mol/L NaCl, and 3.0 mol/L NaCl, respectively. The fraction eluted by 1.5 mol/L NaCl was further fractionated using a Sephacryl S-400/HR column (100 cm × 2.5 cm; GE Healthcare Life Sciences, Piscataway, NJ, USA). The purified polysaccharide MSP (6.25 g) was obtained, concentrated, dialyzed, and freeze-dried.

### 3.4. Preparation of Low-molecular-weight Fractions from MSP by Acid Depolymerization

Low-molecular-weight fractions from MSP were prepared by controlled acid depolymerization. Briefly, the polysaccharide MSP was dissolved in 0.01 mol/L of TFA to a final concentration of 10 mg/mL in a flask, and then, the solution was stirred and heated to 80 °C for 100 min in a water bath. TFA was removed by rotary evaporation through adding methanol; then, the hydrolysate was concentrated and dialyzed in a cellulose membrane (molecular weight cut-off 3500) for 72 h. The concentrated retentate was freeze-dried and fractionated by a Sephacryl S-300/HR column (100 cm × 2.5 cm, GE Healthcare Life Sciences, Piscataway, NJ, USA). Each fraction was tested for total sugar content by the phenol–sulfuric acid method [34]. Finally, six low-molecular-weight fractions were obtained, dialyzed, lyophilized, and designed as MSP-Fs: MSP-F1, MSP-F2, MSP-F3, MSP-F4, MSP-F5, and MSP-F6.

### 3.5. Detemination of Molecular Weight and Purity

The molecular weight and purity of the polysaccharides MSP and MSP-Fs were analyzed by HPGPC on a Shodex OHpak SB-804 HQ column (8.0 mm × 300 mm, Showa Denko K.K., Tokyo, Japan) or Shodex OHpak SB-803 HQ column (8.0 mm × 300 mm, Showa Denko K.K., Tokyo, Japan) [27]. The column calibration was carried out with pullulan standards and eluted with 0.2 mol/L Na_2_SO_4_. First, 20 μL of 1% sample solutions was injected. The molecular weight was estimated by reference to a calibration curve made by pullulan standards (*M*w: 5.9 kDa, 9.6 kDa, 21.1 kDa, 47.1 kDa, 107 kDa, 200 kDa, 344 kDa, and 708 kDa, Showa Denko K.K., Tokyo, Japan).

### 3.6. General Technique

The sulfate contents of MSP and MSP-Fs were assayed by the barium rhodizonic acid method using K_2_SO_4_ as the standard [35]. Protein contents of MSP and MSP-Fs were determined as described by Bradford [36] using bovine serum albumin as the standard. All of the above assays were performed in triplicate. Monosaccharide compositions of MSP and MSP-Fs were determined by reversed-phase HPLC [14]. The identification of sugar was done by comparison with reference sugars (l-rhamnose, l-arabinose, d-xylose, l-fucose, d-glucose, d-mannose, d-glucosamine, d-galactose, d-galacturonic acid, and d-glucuronic acid). The molar ratio of the monosaccharide was calculated based on the ratio of the peak areas of monosaccharide and correspondent monosaccharide standard. Desulfation of MSP was carried out as described by Falshaw and Furneaux [37]. The desulfation product of MSP was designated as MSP-Ds. Methylation analyses of MSP and MSP-Ds were carried out as previous described [38,39]. The identification of partially methylated alditol acetates was performed on the basis of the retention time and its mass fragmentation patterns.

### 3.7. FTIR Spectroscopy Analysis

FTIR spectroscopy of MSP and MSP-Fs were recorded on a Nicolet Nexus 470 spectrometer (Thermo Fisher Scientific, Waltham, MA, USA). The polysaccharide was mixed with KBr powder, ground, and pressed into 1-mm pellets for FTIR measurements in the frequency range of 400–4000 cm^−1^.

### 3.8. NMR Spectroscopy Analysis

The polysaccharides MSP and MSP-Fs were deuterium exchanged by three successive freeze-drying steps in 99% D_2_O, and then dissolved in 0.5 mL of 99.97% D_2_O. ^1^H and ^13^C NMR spectroscopy were performed on an Agilent DDZ 500M NMR spectrometer (Agilent Technologies Co. Ltd., Palo Alto, CA, USA). Acetone was used as internal standard (2.225 ppm for ^1^H and 31.07 ppm for ^13^C). ^1^H–^1^H COSY, ^1^H–^13^C HSQC, ^1^H–^13^C HMBC and ^1^H–^1^H NOESY experiments were also performed [40].

### 3.9. Assay of Anticoagulant Activity In Vitro

Anticoagulant activities in vitro of MSP and MSP-Fs were assayed according to Mourâno et al. [41]. APTT assay was measured as follows: 90 µL of citrated normal human plasma samples and 10 µL of sample solution at concentrations of 0 µg/mL, 10 µg/mL, 25 µg/mL, 50 µg/mL, and 100 µg/mL were incubated at 37 °C for 1 min; then, 100 µL of APTT assay reagent pre-incubated at 37 °C for 10 min was added, and incubated at 37 °C for 2 min. Pre-warmed 100 µL of 0.25 mol/L calcium chloride was then added, and the clotting time was recorded. For PT assay, 90 µL of citrated normal human plasma was mixed with 10 µL of a solution of polysaccharide and incubated at 37 °C for 1 min; then, 200 µL of PT assay reagent that was pre-incubated at 37 °C for 10 min was added, and the clotting time was recorded. For TT assay, 90 µL of citrated normal human plasma was mixed with 10 µL of polysaccharide samples and incubated at 37 °C for 1 min. Then, 200 µL of a TT assay reagent pre-incubated at 37 °C for 10 min was added, and the clotting time was recorded. A saline solution (0.9% NaCl) was used as control in the experiment. Heparin was used for the comparison of the anticoagulant activity of the polysaccharide.

### 3.10. Assessment of Anticoagulant Activity In Vivo

Anticoagulant activities in vivo of MSP and MSP-F4 were assayed with APTT, TT, and PT. In brief, SD rats were divided into eight groups at random (10 rats/group). The rats were anaesthetized by 15% urethane, and then injected with MSP (4 mg/kg, 8 mg/kg, 16 mg/kg), MSP-F4 (4 mg/kg, 8 mg/kg, 16 mg/kg) and heparin (0.5 mg/kg). A saline solution was used as control. After 30 min, the rats were secured in the supine position, and the blood was taken from the abdominal aorta. APTT, TT, and PT assays of the plasma were performed according to the above-mentioned methods.

### 3.11. Assessment of Fibrin(ogen)olytic Property In Vivo

The in vivo fibrin(ogen)olytic properties of MSP and MSP-F4 were determined by FDP, D-dimer, and PAI-1 assays [42,43,44]. Briefly, SD rats were divided into eight groups at random (10 rats/group). The rats were anaesthetized by 15% urethane, and then injected with MSP (5 mg/kg, 10 mg/kg, and 20 mg/kg), MSP-F4 (5 mg/kg, 10 mg/kg, and 20 mg/kg) and urokinase (20,000 U/kg). Saline solution was used as control. After 30 minutes, the rats were secured in the supine position, and the blood was taken from the abdominal aorta. FDP, D-dimer, and PAI-1 levels in the blood were measured using corresponding commercial kits. FDP and D-dimer were assays on a CS-5100 automated blood coagulation analyzer (Sysmex Corporation, Kobe, Japan). PAI-1 was determined on a CA-7000 automated blood coagulation analyzer (Sysmex Corporation, Kobe, Japan).

### 3.12. Assay of Thrombolytic Activity In Vitro

The in vitro thrombolytic activities of MSP and MSP-F4 were measured by clot lytic rate assay [45]. The blood was drawn from the abdominal aorta of SD rats, and placed at room temperature until the big blood clot was finally formed. The clot was washed with saline solution, and the liquid in the surface of the clot was taken away using filter paper. The clot was cut into suitable pieces, weighed, and put into polyethylene tubes, respectively. The tubes were divided into eight groups at random (six clots/group): MSP and MSP-F4 (5 mg/mL, 10 mg/mL, 20 mg/mL), urokinase (100 U/mL) and saline solution groups. A sample (1 mL) was added to the tube, and incubated for 24 h at 37 °C. The residual clot was taken out, and the liquid in the surface of clot was taken away. The wet weight of the residual clot was measured. The clot lytic rate was calculated according to the following equation: clot lytic rate (%) = (1 − Wr/W) × 100, where W is the wet weight of the whole clot, and Wr is the wet weight of the residual clot.

### 3.13. Statistical Analysis

All of the data are representative of at least three independent experiments. Data are presented as means ± standard deviations. Statistical significance was in GraphPad Prism 6 software (La Jolla, CA, USA) using one-way ANOVA with Turkey’s test. *p*-values < 0.05 were considered significant.

## 4. Conclusions

The yield of MSP from dry alga was about 6.25%, which was lower than those of the sulfated polysaccharides from other alga species. MSP was a sulfated rhamnan consisting of →3)-α-l-Rha*p*-(1→ and →2)-α-l-Rha*p*-(1→ residues, with partially sulfate substitutions at the C-2 of →3)-α-l-Rha*p*-(1→ and C-3 of →2)-α-l-Rha*p*-(1→ residues. Six sulfated rhamnan fractions with different molecular weights were obtained by the controlled acid degradation of MSP. The chemical compositions and structures of MSP-Fs are similar to those of the parent MSP. MSP and MSP-F1–MSP-F4 with molecular weights of 24–240 kDa had strong anticoagulant activities. However, a decrease in the molecular weight of MSP-Fs was accompanied by a decrease in the anticoagulant activity, and higher anticoagulant activity requires a molecular weight of over 12 kDa. The molecular weight had a significant effect on the anticoagulant activity of the sulfated rhamnan, and a longer chain was essential to complete thrombin inhibition. MSP and MSP-F4 possessed strong anticoagulant activities in vivo, as well as high fibrin(ogen)olytic and thrombolytic activities. MSP and MSP-F4 have potential as drug or food supplements for human health. An in-depth investigation on the correlation of the anticoagulant activities of sulfated rhamnans with their chemical structures will play a significant role in the understanding of their biological properties. Molecular modification of the active sulfated rhamnans should be done by chemical sulfation, acetylization, and phosphonation. The potential secondary structures of the polysaccharides are formed for biological activity assay. Computational modeling such as artificial neural network is going to be explored for obtaining the desirable polysaccharides with prominent anticoagulant activity in future work.

## Figures and Tables

**Table 1:** Chemical compositions of MSP and MSP-F1–MSP-F6.

Sample	Molecular Weight (kDa)	Sulfate Content (w%)	Monosaccharide Content (mol%)	
			Rha	Xyl
MSP	335	27.32	94.74	5.26
MSP-F1	240	27.15	94.92	5.08
MSP-F2	90	27.30	95.45	4.55
MSP-F3	40	27.88	96.68	3.32
MSP-F4	24	27.61	96.72	3.28
MSP-F5	12	28.32	97.05	2.95
MSP-F6	6.8	28.10	97.14	2.86

**Table 2:** Methylation analyses of MSP and MSP-Ds.

Methylated Alditol Acetate	Molar Ratio (mol%)		Linkage Pattern
	MSP	MSP-Ds	
1,3,5-tri-*O*-acetyl-2,4-di-*O*-methyl-l-rhamnitol	37.52	59.01	→3)-Rha*p*-(1→
1,2,5-tri-*O*-acetyl-3,4-di-*O*-methyl-l-rhamnitol	26.33	29.60	→2)-Rha*p*-(1→
1,2,3,5-tetra-*O*-acetyl-4-*O*-methyl-l-rhamnitol	30.89	6.13	→2,3)-Rha*p*-(1→
1,4,5-Tri-*O*-acetyl-2,3-di-*O*-metyl-xylitol	5.26	nd ^a^	→4)-Xyl*p*-(1→
1,5-Di-*O*-acetyl-2,3,4-tri-O-methyl-xylitol	nd ^a^	5.26	Xyl*p*-(1→

^a^ Not detected.

**Table 3:** ^1^H and ^13^C chemical shifts for MSP-Ds.

Rhamnose Residues ^a^	Chemical Shifts (ppm)					
	H1/C1	H2/C2	H3/C3	H4/C4	H5/C5	H6/C6
A	4.99/103.66	4.20/70.82	3.89/79.55	3.58/73.18	3.79/70.82	1.33/18.23
B	5.07/103.66	4.20/70.82	3.93/79.55	3.57/73.18	3.79/70.82	1.33/18.23
C	5.22/102.45	4.11/79.12	3.98/71.43	3.60/70.79	3.79/70.82	1.33/18.23
D	5.37/102.45	4.25/78.85	4.51/78.85	3.59/70.79	3.79/70.82	1.33/18.23

^a^ A: →3)-α-l-Rha*p*-(1→; B: →3)-α-l-Rha*p*-(1→; C: →2)-α-l-Rha*p*-(1→; D: →2)-α-l-Rha*p*(3SO_4_)-(1→.

**Table 4:** ^1^H and ^13^C chemical shifts for MSP.

Rhamnose Residues ^a^	Chemical Shifts (ppm)					
	H1/C1	H2/C2	H3/C3	H4/C4	H5/C5	H6/C6
A	5.07/103.40	4.20/70.79	3.93/78.29	3.59/73.24	3.79/70.79	1.35/18.16
B	5.25/101.86	4.30/79.47	3.97/70.79	3.61/73.24	3.80/70.79	1.35/18.16
C	5.35/100.75	4.35/79.47	4.47/78.29	3.61/73.24	3.80/70.79	1.35/18.16
D	5.50/100.75	4.72/77.00	4.10/78.29	3.58/73.24	3.79/70.79	1.35/18.16

^a^ A: →3)-α-l-Rha*p*-(1→; B: →2)-α-l-Rha*p*-(1→; C: →2)-α-l-Rha*p*(3SO_4_)-(1→; D: →3)-α-l-Rha*p*(2SO_4_)-(1→. Rha*p*: rhamnopyranose.

**Table 5:** Thrombolytic activities in vitro of MSP and MSP-F4.

Sample	Concentration	Clot Lytic Rate (%)
Control	0 mg/mL	6.66 ± 0.18
MSP	5 mg/mL	13.70 ± 0.62 **^△△^**
	10 mg/mL	20.96 ± 1.91 **^△△^**
	20 mg/mL	34.29 ± 1.68 **^△△^**^□□^
MSP4	5 mg/mL	7.76 ± 0.51
	10 mg/mL	11.88 ± 1.29 **^△△^**
	20 mg/mL	20.79 ± 1.17 **^△△^**
Urokinase	100 U/mL	22.20 ± 1.39 **^△△^**

Significance: **^△△^** *p* < 0.01 vs. the control group; ^□□^ *p* < 0.01 vs. the urokinase group.

## Supplementary Materials

The following are available online at http://www.mdpi.com/1660-3397/16/11/445/s1, Figure S1: IR spectra of MSP and MSP-Fs, Figure S2: NMR spectra of MSP, Figure S3: The main disaccharide units of MSP.
