# Supplementary material for: Anticoagulant Properties of a Green Algal Rhamnan-type Sulfated Polysaccharide and Its Low-molecular-weight Fragments Prepared by Mild Acid Degradation
Source: Mar Drugs. 2018 Nov 12;16(11):445. doi: 10.3390/md16110445 (PMC6266706; doi:10.3390/md16110445)
Supplement: Supplementary file 1 [file marinedrugs-16-00445-s001.pdf]

SUPPLEMENTARY FIGURE LEGENDS

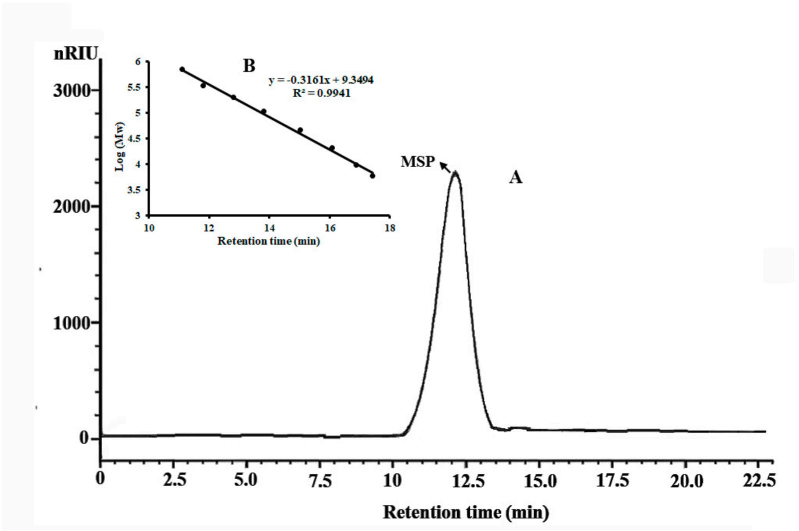

Figure S1. IR spectra of MSP and MSP-Fs.

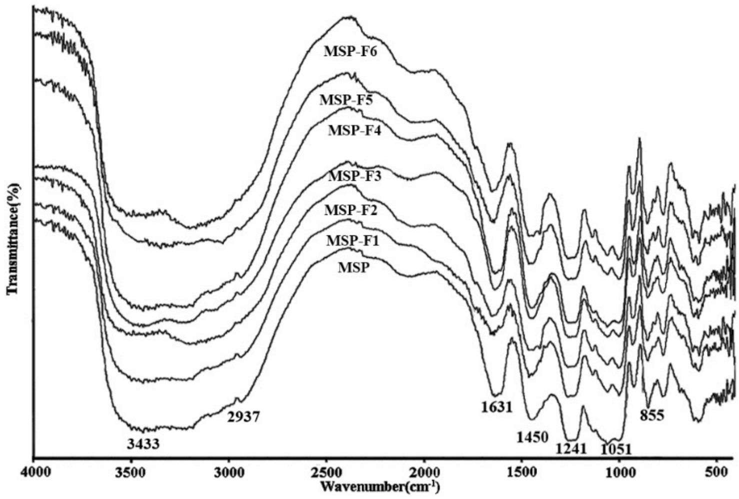

Figure S2. HPGPC chromatogram of MSP and the standard curve of molecular weight. (A) HPGPC chromatogram of MSP; (B) the standard curve of molecular weight.

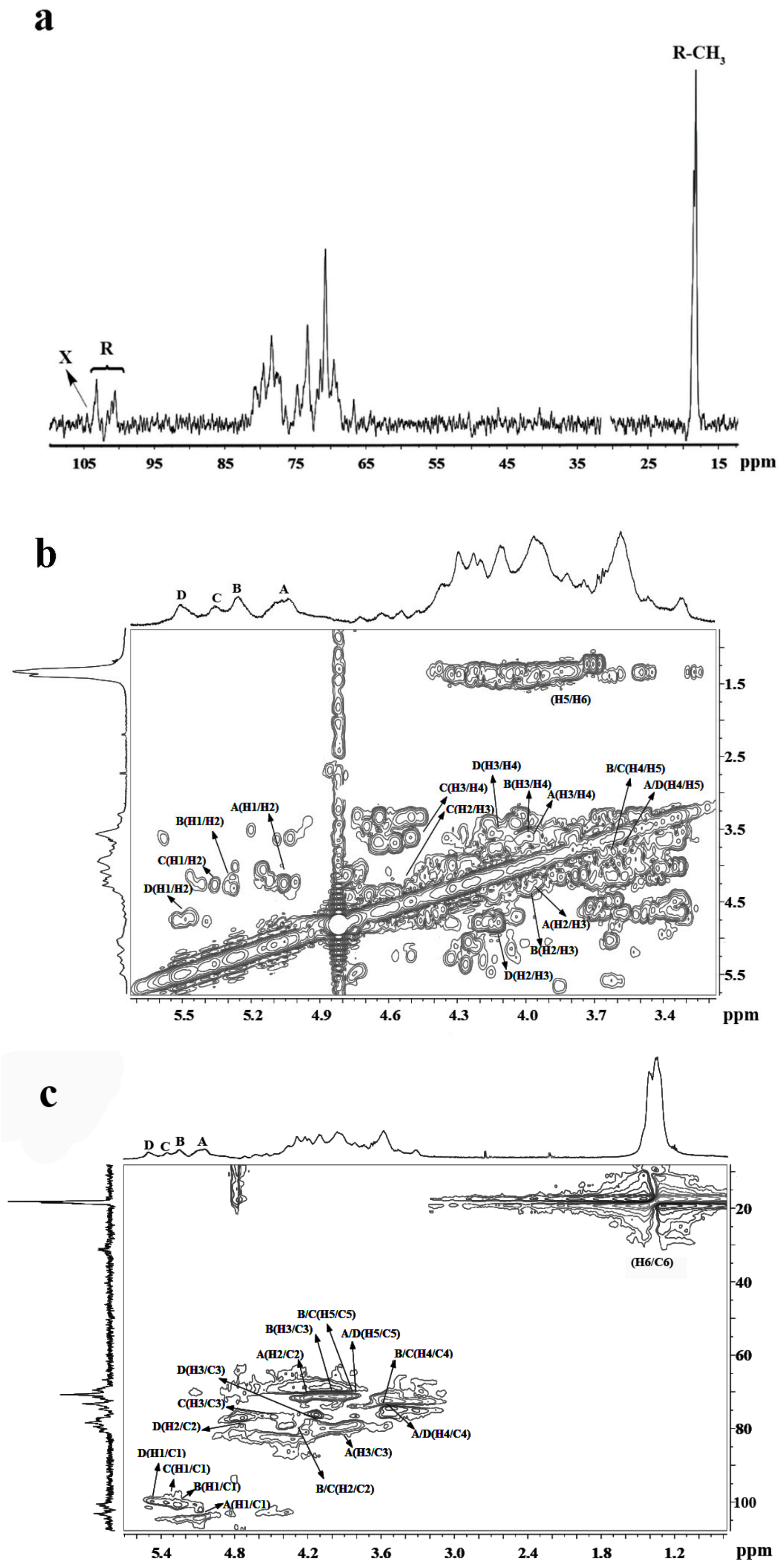
a
R-CH3
X
R
105 95 85 75 65 55 45 35 25 15 ppm
b
D C B A
(H5/H6)
D(H3/H4)
C(H3/H4)
C(H2/H3)
B(H3/H4)
A(H3/H4)
B/C(H4/H5)
A/D(H4/H5)
A(H1/H2)
B(H1/H2)
C(H1/H2)
D(H1/H2)
A(H2/H3)
B(H2/H3)
D(H2/H3)
1.5 2.5 3.5 4.5 5.5
5.5 5.2 4.9 4.6 4.3 4.0 3.7 3.4 ppm
c
D C B A
(H6/C6)
B/C(H5/C5)
B(H3/C3)
A/D(H5/C5)
A(H2/C2)
B/C(H4/C4)
D(H3/C3)
C(H3/C3)
D(H2/C2)
A/D(H4/C4)
A(H3/C3)
D(H1/C1)
C(H1/C1)
B(H1/C1)
A(H1/C1)
B/C(H2/C2)
20 40 60 80 100
5.4 4.8 4.2 3.6 3.0 2.4 1.8 1.2 ppm

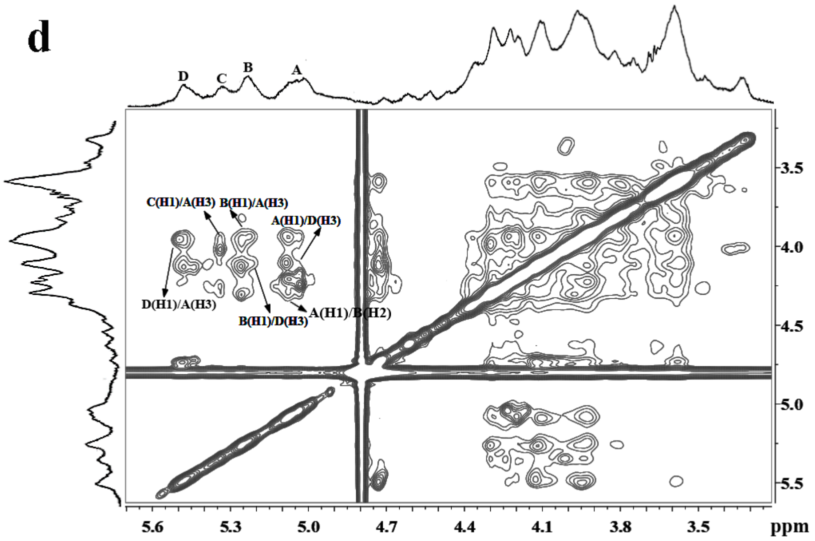

Figure S3. ^{13}C NMR and 2D NMR spectra of MSP. Spectra were performed on an Agilent DD2 500M NMR spectrometer. Chemical shifts are referenced to internal acetone at 2.225 ppm for ^{1}H and 31.07 ppm for ^{13}C. (a) ^{13}C NMR spectrum; (b) ^{1}H–^{1}H COSY spectrum; (c) ^{1}H–^{13}C HSQC spectrum; (d) ^{1}H–^{1}H NOESY spectrum. A–D correspond to →3)-α-L-Rha*p*-(1→, →2)-α-L-Rha*p*-(1→, →2)-α-L-Rha*p*(3SO_4)-(1→ and →3)-α-L-Rha*p*(2SO_4)-(1→, respectively. Rha*p*: rhamnopyranose; R: rhamnose; X: xylose.

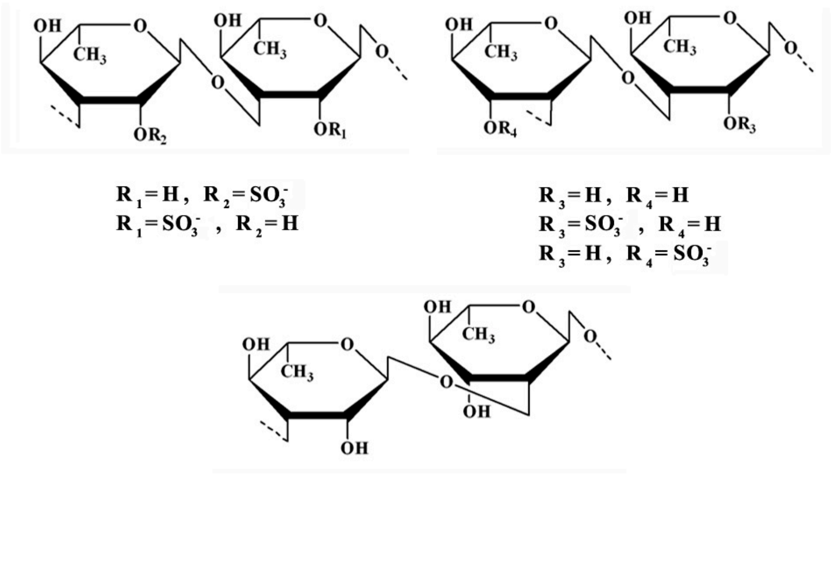

Figure S4. Structures of the main repeating disaccharides of MSP.
